# Nucleosome Positioning around Transcription Start Site Correlates with Gene Expression Only for Active Chromatin State in Drosophila Interphase Chromosomes

**DOI:** 10.3390/ijms21239282

**Published:** 2020-12-05

**Authors:** Victor G. Levitsky, Tatyana Yu. Zykova, Yuri M. Moshkin, Igor F. Zhimulev

**Affiliations:** 1 Department of System Biology, Institute of Cytology and Genetics, 630090 Novosibirsk, Russia; moshkin@bionet.nsc.ru; 2 Department of Natural Sciences, Novosibirsk State University, 630090 Novosibirsk, Russia; 3 Department of the Structure and Function of Chromosomes, Institute of Molecular and Cellular Biology, 630090 Novosibirsk, Russia; vatolina@mcb.nsc.ru

**Keywords:** nucleosome arrangement, chromatin landscape, expression level, breadth of expression, 5′-regulatory region, tissue-specific and silent genes, housekeeping and widely expressed genes

## Abstract

We analyzed the whole-genome experimental maps of nucleosomes in *Drosophila melanogaster* and classified genes by the expression level in S2 cells (RPKM value, reads per kilobase million) as well as the number of tissues in which a gene was expressed (breadth of expression, BoE). Chromatin in 5′-regions of genes we classified on four states according to the hidden Markov model (4HMM). Only the Aquamarine chromatin state we considered as Active, while the rest three states we defined as Non-Active. Surprisingly, about 20/40% of genes with 5′-regions mapped to Active/Non-Active chromatin possessed the minimal/at least modest RPKM and BoE. We found that regardless of RPKM/BoE the genes of Active chromatin possessed the regular nucleosome arrangement in 5′-regions, while genes of Non-Active chromatin did not show respective specificity. Only for genes of Active chromatin the RPKM/BoE positively correlates with the number of nucleosome sites upstream/around TSS and negatively with that downstream TSS. We propose that for genes of Active chromatin, regardless of RPKM value and BoE the nucleosome arrangement in 5′-regions potentiates transcription, while for genes of Non-Active chromatin, the transcription machinery does not require the substantial support from nucleosome arrangement to influence gene expression.

## 1. Introduction

Nucleosomes are the basic packaging units of chromatin. Their positioning has a critical impact on the regulation of transcription. A wide variety of chromatin proteins can affect nucleosome positions [1,2]. Besides the competitive or cooperative binding of other proteins [3], ATP-dependent chromatin-remodeling complexes (remodelers) [4,5] overrule intrinsic DNA sequence preferences of histone octamers [6]. Thus, these preferences make only a modest contribution to the pattern of nucleosome positioning in vivo (≈20%) [7,8], a stronger impact comes from the pattern of histone variants and modifications and the opportunities of a number of non-histone proteins to interact with DNA [2,9].

Intrinsic DNA sequence preferences due to the thermodynamic costs associated with wrapping stiff DNA around the histone octamer influence on nucleosome positions in vivo [2,9]. A wide variety of chromatin proteins can affect nucleosome positions, most notably ATP-dependent chromatin-remodeling complexes [1,5]. Nucleosome positioning have a critical impact on regulation of transcription. The combination of DNA context dependent and chromatin protein-influenced positioning fine-tunes the accessibility of gene regulatory regions and their competence for transcription machinery [10] affecting both initiation and elongation of transcription.

Experimental data on nucleosome positioning in genomes come from a number of related techniques [1,10,11]. For example, the digestion of chromatin with micrococcal nuclease (MNase) [11] is used to detect nucleosome positions. It is generally accepted that promoters are nucleosome-depleted; the region of diminished nucleosome occupancy observed at proximal promoter is referred to as nucleosome-depleted region (NDR) [1]. In most genes NDRs are flanked by two positioned nucleosomes (positions Nuc−1 and Nuc+1 relative TSS) [2,12,13,14,15,16,17]. These nucleosomes only for expressed genes are highly phased relative to their transcription start sites (TSSs) [17]. Analysis of various species supported this concept [8,18,19,20,21]. However, besides the expression level, many other factors could influence nucleosome arrangement around TSS. For example, in comparison to wild type, yeast strains carrying mutations in genes with known or suspected roles in nucleosome biology revealed the significant change of nucleosome arrangement [22]. We expect that full genomic separation of genes into groups [23,24] according to inherent chromatin environment influences gene activity and nucleosome arrangement.

Maps of chromatin states were a consequence of recent advances in experimental and bioinformatics approaches for genome analysis. First, we earlier developed an approach to simultaneously map the interband material cytologically and in the genome using transposon insertion tags [25]. This allowed exact localization of insertion sites both on cytological and physical maps. Previously, we showed that polytene chromosomes and interphase chromosomes from dividing cells display identical organization [24,26,27]. Namely, interbands from polytene chromosomes and the corresponding DNA sequences from cell line chromosomes share similar features in terms of localization of open chromatin-type proteins. Second, efforts of the modENCODE project [28] have produced genome-wide profiling data for many proteins that specifically localized to bands or interbands in interphase chromosomes [23,24,27,29].

These advances in cytology and bioinformatics allowed partitioning the genome into four chromatin types referred to as Aquamarine, Lazurite, Malachite, and Ruby [23,30]. We have earlier shown that genes referred to Aquamarine and Ruby types had notably different levels and patterns of expression. Namely, the median expression level of genes whose 5′-ends were located in Aquamarine chromatin type was substantially higher than that for Ruby type. Similarly, the mean number of tissues for “interband” Aquamarine genes was substantially higher than that for “band” Ruby genes. Hence, the Aquamarine chromatin was interpreted as open, i.e., it harbored regulatory regions of active genes. On the contrary, Ruby chromatin contained mostly tissue-specific genes, intergenic spacers, and introns. Two remaining types respected to gene bodies (Lazurite) and intermediate domains between other three types of chromatin (Malachite) [23,24,30]. All of this permits to carry out an analysis of localization and activity of genes as well as chromatin states and interphase polytene chromosome structures. In the current study we further classified four states [23,30] into “Active” (Aquamarine) and all the rest “Non-Active” (i.e., at most moderately active Lazurite/Malachite and completely non-active Ruby).

The canonical nucleosome arrangement Nuc–1/NDR/Nuc+1 is typical for housekeeping genes; stress-responsive genes have noncanonical nucleosome arrangement due to delocalized, i.e., uniformly distributed nucleosomes [9]. Partial/complete digestion of Drosophila chromatin with high/low concentrations of MNase revealed whole-genome maps of MNase sensitive/resistant types of nucleosomes, respectively [21]. We performed analysis of these maps and classified all genes according to expression level and the chromatin state of promoter DNA and found relatively large fractions of “silent” genes and expressed genes referred to Active and Non-Active chromatin environment, respectively. It was found that the regular/fuzzy nucleosome arrangement in promoters referred to Active/Non-Active chromatin states is conserved, irrespective to either the expression level or the number of expressed tissues.

## 2. Results

### 2.1. Gene Classification by Chromatin States and Expression Measures

We proceeded from the earlier analysis of one cell line [21] to the analysis of multiple tissues. We analyzed 13,574 protein-coding genes from FlyBase with RNA-seq profile for 29 tissues [31] and cell types [32], and unique chromosome position of TSS (see Section 4).

We classified all genes according to RPKM value (reads per kilobase million) in S2 cells [30] on three classes:

- Silent (RPKM = 0, 6972 genes);
- Modest (1 ≤ RPKM ≤ 25, 3718);
- High (RPKM > 25, 3584).

We computed the breadth of expression (BoE) as the number of tissues in which a gene was expressed. We considered genes satisfied the criterion RPKM > 3 to compute BoE. We classified genes by BoE on three classes:

- Tissue-specific (BoE ≤ 6, 5534 genes);
- Modest (6 > BoE ≤ 24, 4278);
- Constitutive (BoE >24, 3942).

To incorporate the chromatin landscape in the analysis we considered the whole-genome 4-state fragmentation model 4HMM [23,30]. We computed the number of genes that were masked by certain state at a specific distance from TSS. For any state a number of genes remained approximately constant within the region (−300; +200) around TSS (Figure S1A). According to 4HMM the Aquamarine chromatin below will be referenced to as “Active”, and the remaining three states as “Non-Active”. Hence, below we consider 6565/6209 genes with this region entirely encompassed by the Active domains (“Active” genes) or any other domain (“Non-Active” genes).

**Figure 1:**
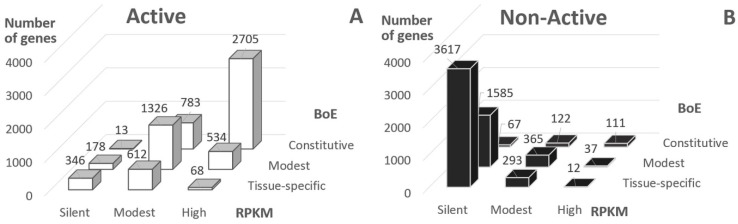
Classification of genes according to expression level (RPKM, reads per kilobase million) and breadth of expression (BoE). BoE was computed for 29 tissues; RPKM values respected to S2 cells; panels (**A**,**B**) show “Active” and “Non-Active” genes, these classes respect to Aquamarine and Lazurite/Malachite/Ruby chromatin states according to 4HMM [23,30] (see Section 4).

The distribution of Active genes into classes of RPKM value and BoE demonstrated that the majority of genes had at least modest BoE or RPKM (81.5%, Figure 1A, RPKM > 0 or BoE > 6). On the contrary, the majority of Non-Active genes were silent or tissue-specific (58.3%, Figure 1B). Hence, about 20% of Active genes were either silent or tissue-specific, and about 40% of Non-Active genes revealed either positive RPKM value or modest/high BoE.

### 2.2. Irrespective to Expression Measures, Nucleosome Arrangement Is Conservative for Active Genes

We appealed to experimental data on the mapping of resistant/sensitive nucleosomes (see Section 4, [21]) to explain the diverse classification of genes by the chromatin state and expression measures (Figure 1). We computed the number of nucleosome sites mapped for various RPKM/BoE classes in the region (−300; +200) relative to TSS for Active and Non-Active genes.

**Figure 2:**
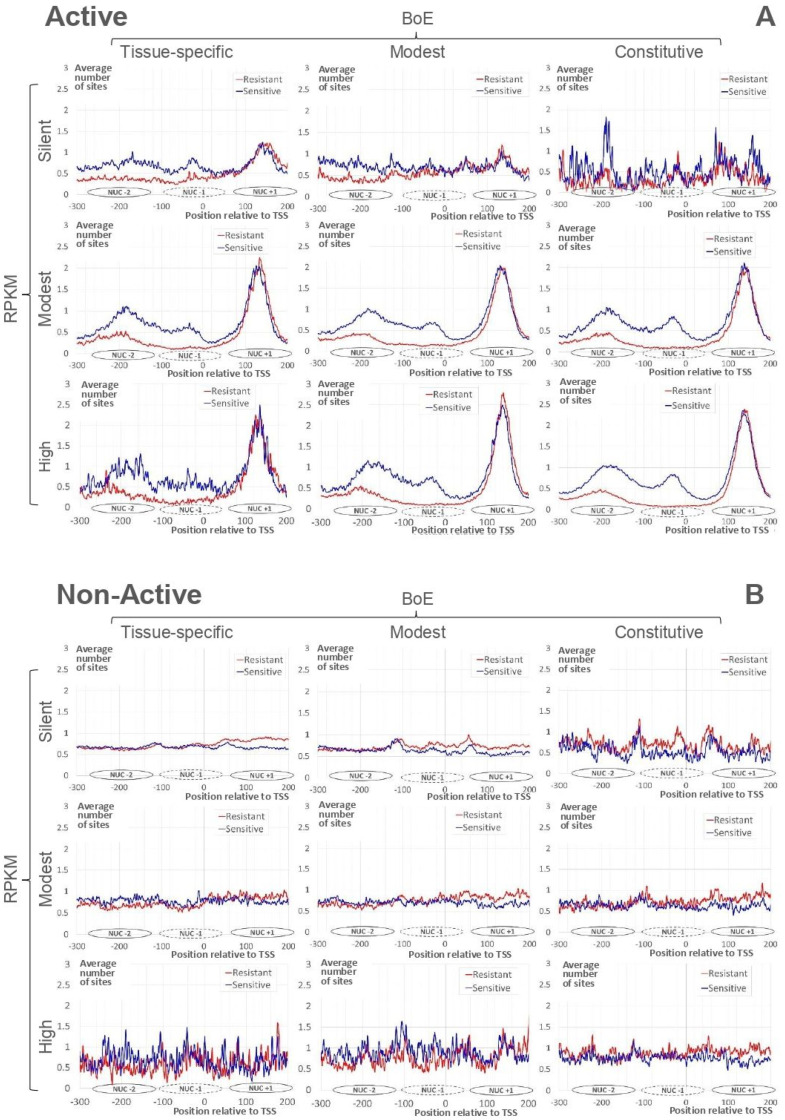
Average numbers of mapped nucleosome sites for “Active” (**A**) and “Non-Active” (**B**) genes (see Section 4). Red/blue lines mark resistant/sensitive nucleosomes [21]. X axes denote the position relative to TSS. Y axes show the moving mean over a window of 3 nt for average number of nucleosome sites. The headers of columns/rows mean the classification of genes by BoE/RPKM. Ovals near X axes denote approximate positions of nucleosomes upstream (Nuc−2, Nuc−1) and downstream (Nuc+1) TSS.

Figure 2A demonstrates the profiles of the average number of resistant and sensitive nucleosome sites for Active genes. The strong nucleosome positioning site downstream TSS (the nucleosome center position +135, Nuc+1) is clearly visible for all datasets. The slight blurring of the peak we found only for “silent” genes. According to the previous observation [21], profiles for resistant and sensitive nucleosome sites show the notable difference in the region upstream TSS. The depletion of resistant nucleosome sites just upstream TSS are clear for all datasets besides the “silent”. In the upstream region, peaks for centers of sensitive nucleosomes are located in positions −35 (Nuc−1) and −185 (Nuc−2) for genes with the modest/high RPKM. The majority of silent Active genes were tissue-specific (346, Figure 1A). These genes revealed the arrangement similar to that of genes with the modest/high RPKM.

Figure 2B shows profiles of the average number of resistant/sensitive nucleosome sites for Non-Active genes. These profiles are substantially divergent from those of Active genes (Figure 2A). Namely, we did not find

- the strong nucleosome formation sites downstream TSS;
- the depletion of resistant nucleosome sites upstream TSS;
- the regular ladder of sensitive nucleosomes upstream TSS.

Note that peaks at positions Nuc−2, Nuc−1, and Nuc+1 are completely absent in genes with modest/high RPKM or in “modest BoE”/constitutive genes.

Thus, for Non-Active genes respecting modest/high RPKM or “modest BoE”/constitutive classes (Figure 2B), we did not detect the arrangement revealed above for Active genes (Figure 2A). The comparison of profiles for Active/Non-Active genes (Figure 2) revealed that irrespective to RPKM/BoE Active genes possessed the common pattern of nucleosome arrangement that was absent in Non-Active genes.

### 2.3. Correlations between Expression Measures and Nucleosome Positioning

**Figure 3:**
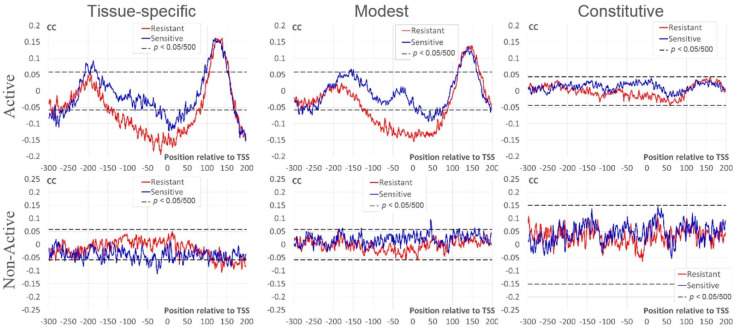
Correlation coefficients (CC) between the expression level (RPKM) and the number of mapped nucleosomes. The top/bottom rows denote datasets of Active/Non-Active genes (see Section 4). The left, middle, and right columns mark intervals of the expression breadth (BoE). X axes show the position relative to TSS, Y axes denote the moving mean of CC over a window of three nt. Red/blue lines denote resistant/sensitive nucleosomes [21]. Dashed black lines mark the threshold of Bonferroni corrected *p*-value for CC (*p* < 0.05/500).

In this section, we study dependencies between the number of mapped nucleosome sites around TSS and expression measures RPKM/BoE. This analysis was performed separately for Active and Non-Active genes. We applied Kendall’s rank correlation coefficients (CC) (see Section 4) to reveal significant dependencies. Figure 3 shows profiles of CC between the RPKM value and the number of resistant/sensitive sites for three classes of BoE. We found several differences between profiles of CC in Active and Non-Active genes. Thus, for Active genes we found:

- strongly significant negative CC for resistant nucleosomal sites around TSS [−120; +80] for tissue-specific and modest; moderate negative CC for sensitive nucleosomal sites for tissue-specific [−30; +50] and modest [+10; +70];
- strongly significant positive CC downstream TSS [+70; +200] for tissue-specific and modest;
- moderately significant positive CC for sensitive nucleosomal sites upstream TSS in tissue-specific [−210; −180]) and modest [−160; −150].

In Non-Active genes the 1st and 3rd trends are not observed; the 2nd trend is even opposite for tissue-specific and is absent for the rest genes.

Constitutive Active and Non-Active genes did not show significant correlation (Figure 3). However, the pattern of Active genes downstream TSS is similar to that of genes with lower BoE ([+130; +160] in Figure 3). As we expected, the tendency to a positive correlation is completely absent in this region in constitutive Non-Active genes.

Figure S2 shows profiles of CC between BoE and the number of resistant/sensitive nucleosomal sites for three classes of RPKM value. Though correlations for the BoE, in general, are less significant compared to those for the RPKM value (Figure 3), the pattern for Active genes still is clearer than that for Non-Active genes.

## 3. Discussion

The development of whole-genome maps of chromatin states [23,33,34,35] and the availability of whole transcriptome profiling data has [31,32,36] created a rich resource for bioinformatics analyses. Earlier, Filion et al. [33] suggested that the chromatin states act as guides that helped to target DNA binding factors to specific regions of the genome, even though the cognate binding motifs were broadly distributed. In the current study, we supposed that this statement refers to histones too. Particularly, the chromatin state may also guide the gene expression through the nucleosome positioning. Hence, for various chromatin states we analyzed the association between the expression measures and the number of mapped nucleosome sites in specific position relative to TSS. The gene expression we measured with the expression level in S2 cells (RPKM value) and the breadth of expression (BoE, the number of expressed tissues among 29 tissue types annotated in FlyBase database [32]). We analyzed the distribution of domains of chromatin states relative TSS for the 4-state [23,30] and 5-state [33] maps of chromatin states. We selected the criterion of entire overlap of the region (−300; +200) relative TSS with domains of certain chromatin state and partitioned all genes (Figure S1) on “Active” and “Non-Active” classes. These datasets we further classified by RPKM value and BoE (Figure 1 and Supplementary Figure S3). Although, the Active genes generally had higher RPKM and BoE than Non-Active genes,

- about 20% of Active genes were either silent or tissue-specific;
- about 40% of Non-Active genes had at least modest RPKM or BoE.

Then, along the length of promoter, we computed the profiles of the average numbers of resistant/sensitive nucleosome sites for Active and Non-Active genes and categorized them by RPKM value and BoE. Notably, these subsets revealed clear diversity in the nucleosome arrangement around TSS (Figure 2 and Supplementary Figure S4). We concluded that the regular nucleosome positioning is the specific feature of the Active chromatin state.

Next, we tested the significance of correlations between the number of resistant/sensitive nucleosomal sites [21] and RPKM/BoE for Active and Non-Active genes in various promoter positions. We performed this analysis separately for every class of RPKM/BoE (Figure 1). We confirmed that Active and Non-Active genes had substantially different dependencies between the number of nucleosomal sites and the expression level. Whereas Active genes revealed the conservative pattern of significant dependencies in various positions of promoter, Non-Active genes did not have this pattern (Figure 3 and Supplementary Figure S5). The similar trend we found for the breadth of expression (Supplementary Figures S2 and S6), i.e., the conservative pattern of nucleosome positioning around TSS, is more prominent for Active genes than for Non-Active genes.

Our conclusions are in agreement with a number of recent reports:

- It was estimated [37] that almost half of the human genome contained regular arrays of nucleosomes that enriched in active chromatin domains. Since the presence of transcription factor (TF) binding sites (BS) strongly supports gene expression, the analysis examined the positioning of nucleosomes around these BS using ChIP-seq data for 35 different TFs. As a result, TF occupancy was closely related to the strength of nucleosome positioning.
- The genome-wide location of nucleosomes during zebrafish embryogenesis indicated that well-positioned nucleosome arrays appeared on thousands of promoters during genome activation [38]. It is important to remember that both models [23,30,33] of chromatin states were based on whole genome profiles of a number of non-histone proteins, i.e., the pattern of BS of these proteins should be different in Active in Non-Active genes.
- The recent analysis of chromatin folding for various epigenetic states of chromatin [39] supported the importance of chromatin state annotation for analysis of genome-wide nucleosome organization. In particular, the classification of genomic domains in Drosophila cells into transcriptionally active, inactive, or Polycomb-repressed states revealed distinct chromatin organizations for each state.

Earlier [21], genes ordered by their expression levels showed marked dichotomy, with highly expressed genes exhibiting much more pronounced nucleosome positioning around the TSS. Based on our analysis, we propose two distinct mechanisms associated with mapping of gene promoter to domains of Active or Non-Active chromatin states. Regardless of the expression level or breadth of expression, the nucleosome positioning in the promoter:

- for Active state potentiates transcription, i.e., a more regular nucleosome arrangement in promoter points to higher expression level and higher number of expressed tissues;
- for Non-Active state does not influence gene expression, i.e., the expression level/breadth and the nucleosome positioning are independent.

## 4. Materials and Methods

### 4.1. Whole Genome Data Analysis

We took the nucleosome mapping data [21] (GEO AC GSE69336) as the whole-genome profiles for chromosome arms 2L, 2R, 3L, 3R, and X. These data included the results for MNase-sensitive/resistant nucleosome positioning sites (low/high concentration of MNase) for S2 cells.

We used the dataset of RNA-seq data from the modENCODE database [28], FB2013_03 release of FlyBase [40]. This table represents expression levels (RPKM values) for 29 tissues (FBlc0000206) [31] and S2 cells (FBlc0000260) [32]. We considered 13,574 protein-coding genes from the FlyBase database [41] with distinct TSS and present RNA-seq data (Supplementary Table S1). We applied the threshold RPKM = 3 for all tissues to compute the breadth of expression (BoE), the number of tissues with RPKM values exceeded the threshold.

We applied the 4-state chromatin map (4HMM) [23,30]. We considered the mapping of 5′-regulatory region of genes to domains of Aquamarine (or Active) state as the marker of an active gene. In particular, genes possessing region (−300; +200) around TSS entirely mapped within domains of Active and any other states (Lazurite, Malachite, and Ruby) we referred to as Active and Non-Active, respectively. Additionally, we considered the 5-state chromatin map [33] (the Yellow/Red and Green/Blue/Black states defined Active and Non-Active genes, respectively).

### 4.2. Statistical Analysis

We computed Kendall’s rank correlation coefficients (CC) to estimate the significance of correlations between the expression measure (BoE or RPKM) and the number of nucleosomes mapped in a specific position. To estimate the significance of Kendall’s rank CC we applied the normal approximation [42] with the mean zero and the variance *D:*

$D=\frac{2\times \left(2\times N+5\right)}{9\times N\times \left(N-1\right)}$, here *N* denotes the sample size.

## 5. Conclusions

We took the whole-genome map of chromatin states and referred “Active” and “Non-Active” genes according to the overlap of their 5′-regulatory regions with domains of Active and Non-Active chromatin states. Our analysis confirmed that classification of all genes according chromatin states allowed deducing the better explanation for the influence of nucleosome positioning on gene transcription activity. We found that irrespective to the expression level or the number of expressed tissues:

- Active genes demonstrated clear pattern of nucleosome arrangement around TSS; the regular nucleosome positioning correlates with the expression level/breadth;
- Non-Active genes did not show such clear specificity in nucleosome positioning; the correlation between the regular nucleosome positioning and expression level or breadth either is absent, not conservative, or shows even the opposite significant trend compared to that of Active genes.

The analysis of 5-state model of Filion et al. [33] supported these conclusions (Supplementary Figures S1B and S3–S6).

## Supplementary Materials

Supplementary materials can be found at https://www.mdpi.com/1422-0067/21/23/9282/s1.

## Abbreviations

4HMM	Four state hidden Markov model
BoE	Breadth of expression
MNase	Micrococcal nuclease
Nuc	Nucleosome
NDR	Nucleosome-depleted region
RPKM	Reads per kilobase million
TSS	Transcription start site
