# Supplementary material for: Nucleosome Positioning around Transcription Start Site Correlates with Gene Expression Only for Active Chromatin State in Drosophila Interphase Chromosomes
Source: Int J Mol Sci. 2020 Dec 5;21(23):9282. doi: 10.3390/ijms21239282 (PMC7730318; doi:10.3390/ijms21239282)
Supplement: Supplementary file 1 [file ijms-21-09282-s001.zip › ijms-1006284-supplementary/ijms-1006284-supplementary.pdf]

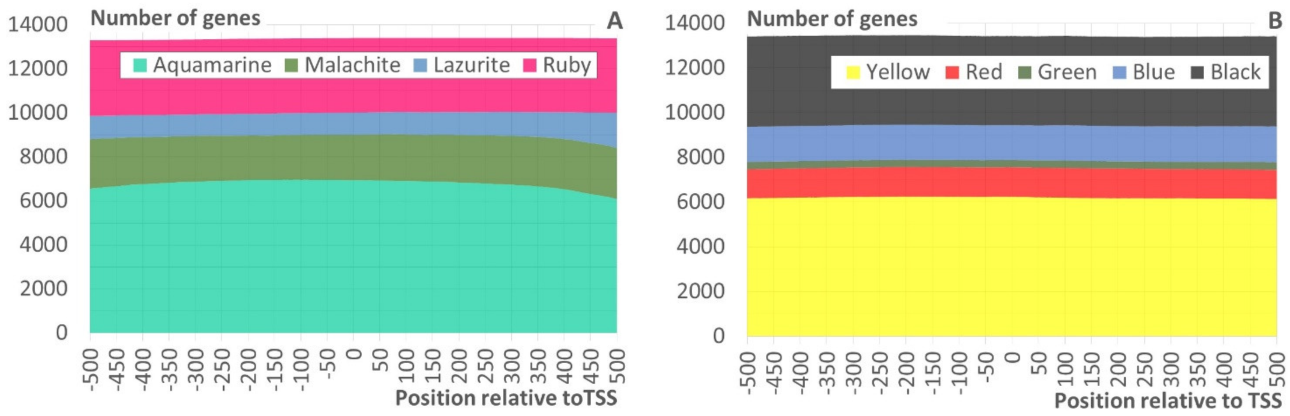

Figure S1. Distribution of domains of various chromatin states relative to TSS. (**A**) – 4-state model of Lazurite, Aquamarine, Malachite And Ruby states [23,30]. (**B**) – 5-state model of Yellow, Red, Green, Blue and Black states [33]. Position relative to the TSS is plotted on the X axis. Y axis denotes the number of genes that were marked by a specific chromatin type at a certain position relatively to the TSS.

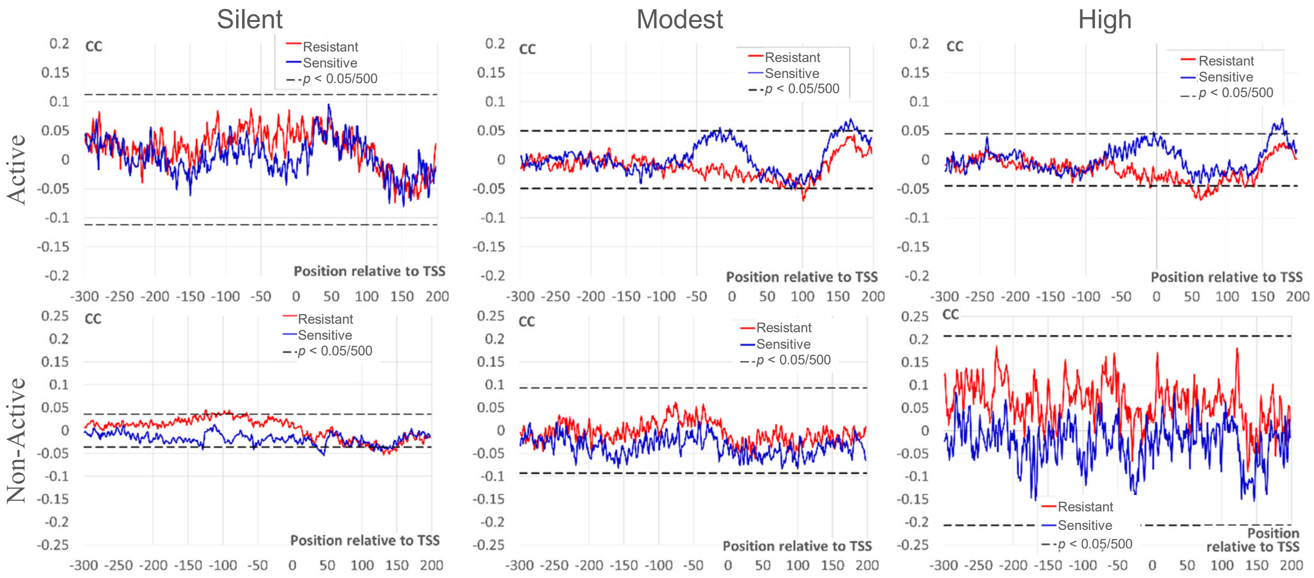

Figure S2. Correlation coefficients between BoE and the number of mapped nucleosomes. The top/bottom rows denote datasets of Active/Non-Active genes (see Materials and Methods). The left, middle and right columns mark intervals of the expression level (RPKM). Axes X show the position relative to TSS, axes Y imply the moving mean for Kendall's rank CC over a window of three nt. Red/blue lines denote resistant/sensitive sites [21]. Dashed black lines mark the threshold of Bonferroni corrected p-value for CC ($p < 0.05/500$). Genes were classified according to the chromatin map [23,30] (see Materials and Methods).

Among silent genes, only Non-Active genes revealed moderately significant positive CC for resistant sites, [−120; −80]. Active genes with modest/high RPKM values revealed moderately significant positive CC of sensitive sites just upstream and downstream TSS. Note that respective regions [−20; +1] and [+160; +180] respect to the centers of nucleosomes Nuc−1 and Nuc+1. The linker region [+50; +85] between these nucleosomes shows the negative correlation of the number of resistant sites (high RPKM). Non-Active genes with modest/high RPKM do not show any significant correlations.

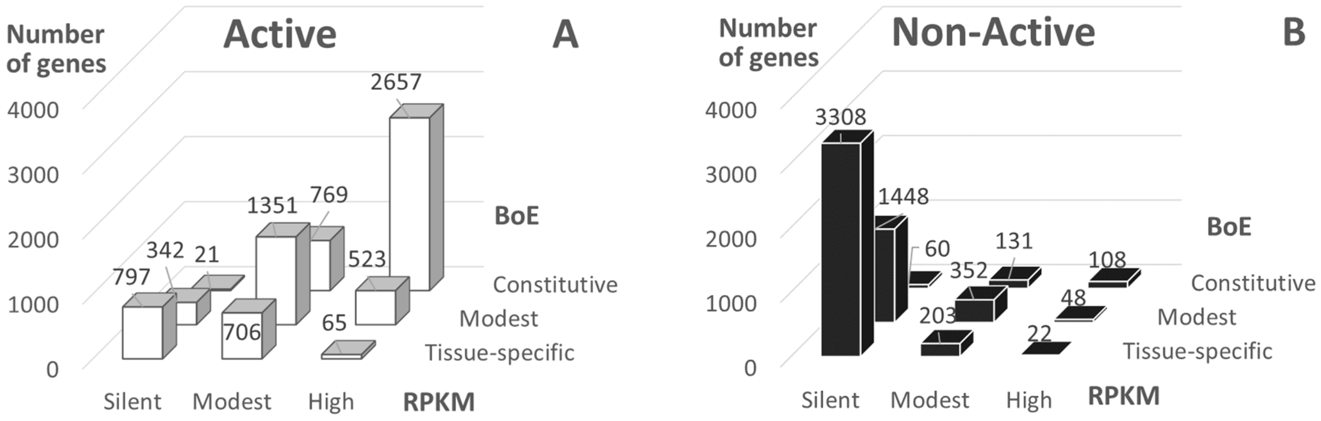

Figure S3. Classification of genes according to expression level (RPKM) and breadth of expression (BoE). BoE was computed for 29 tissues; RPKM values respected to S2 cells; panels (**A**) and (**B**) show Active and Non-Active genes classified according to the chromatin map [33] (see Materials and Methods).

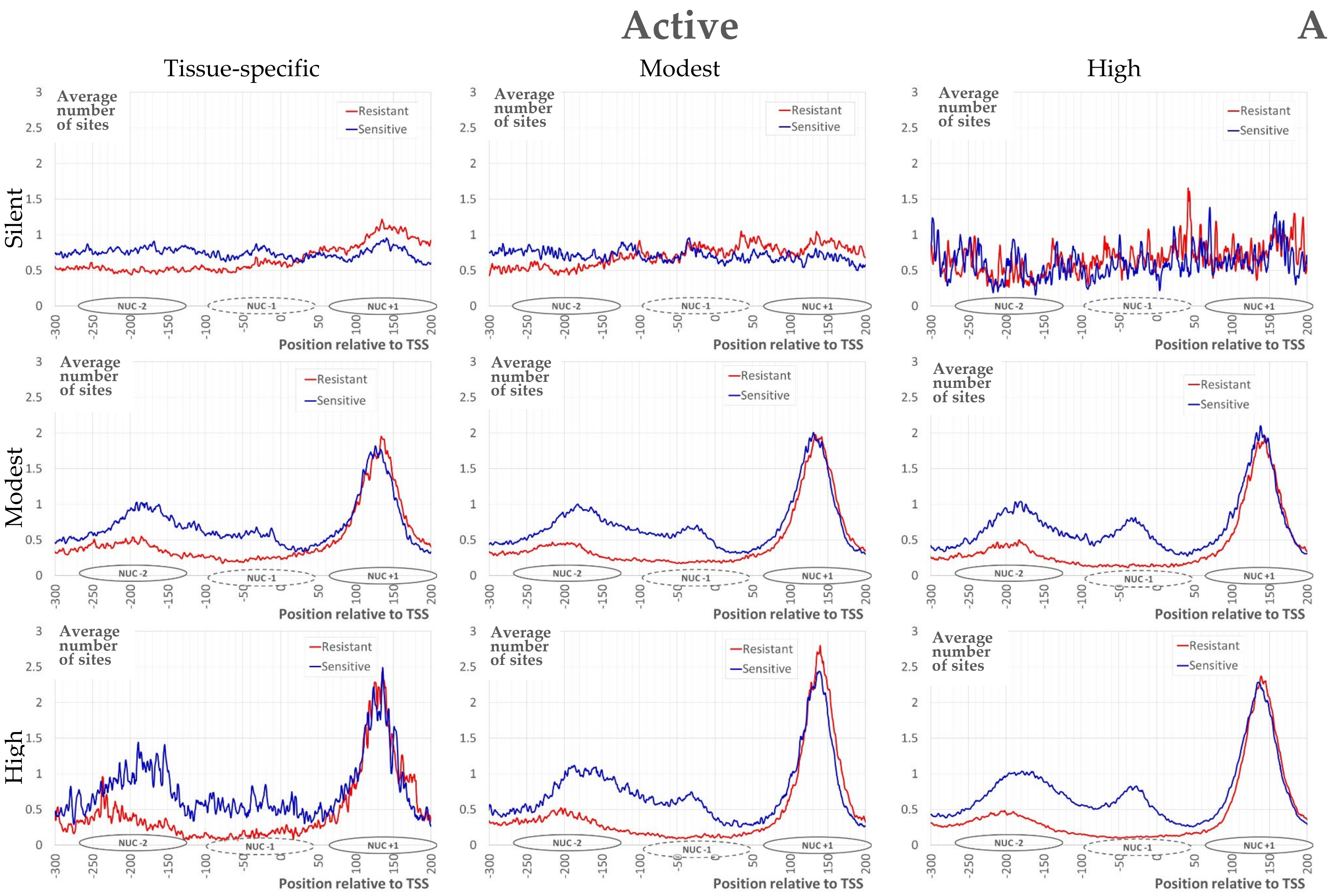
Active
A
Tissue-specific
Modest
High
Silent
Modest
High
Average number of sites
Resistant
Sensitive
NUC -2
NUC -1
NUC +1
Position relative to TSS

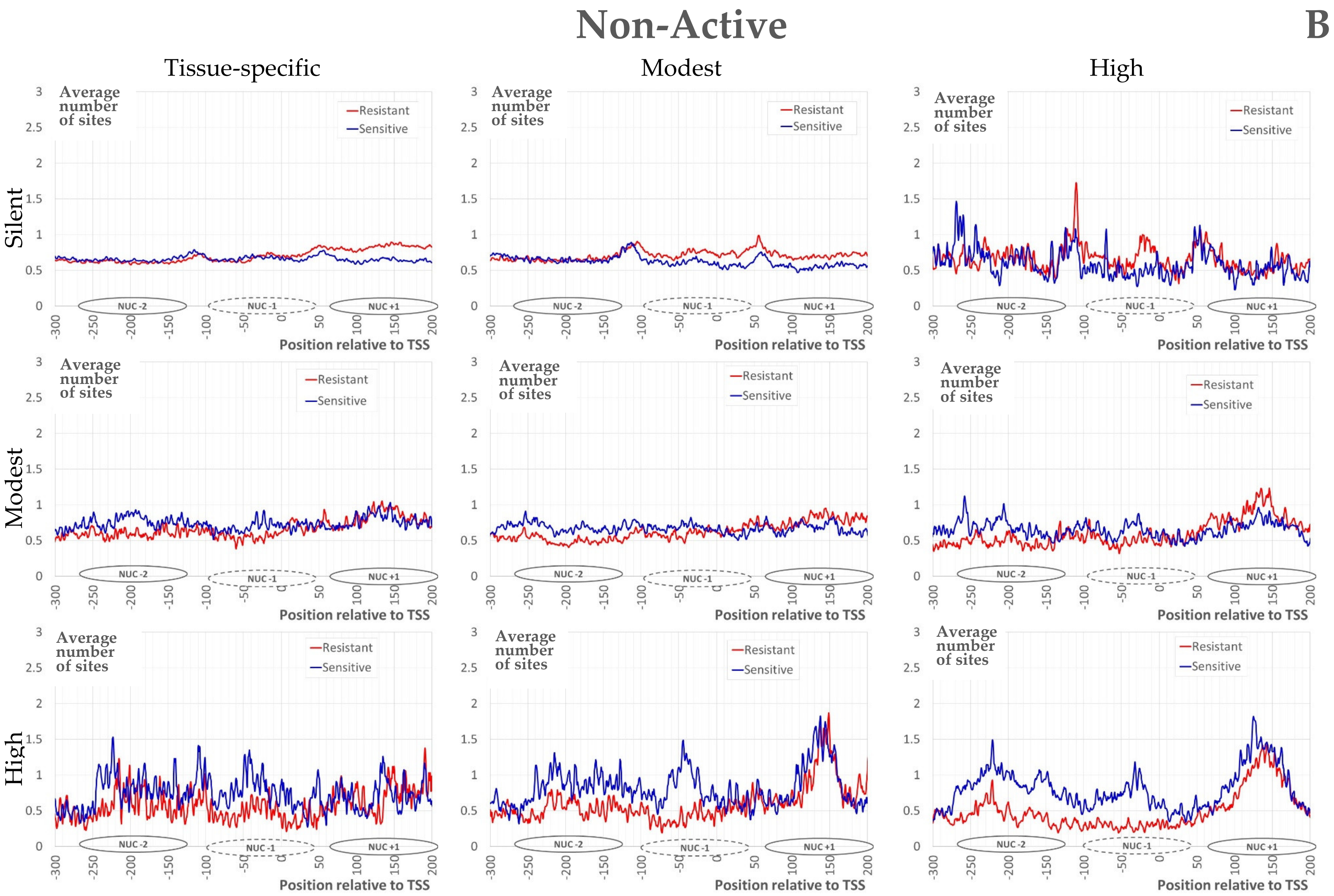

Non-Active
B
Tissue-specific
Modest
High
Silent
Modest
High
Average number of sites
Resistant
Sensitive
NUC -2
NUC -1
NUC +1
Position relative to TSS

Figure S4. Average numbers of mapped nucleosome sites for Active (**A**) and Non-Active (**B**) genes classified according to the chromatin map [33] (see Materials and Methods). Red/blue lines mark resistant/sensitive nucleosomes [21]. Axes X denote the position relative to TSS. Axes Y show the moving mean over a window of 3 nt for average number of nucleosome sites. The headers of columns/rows mean the classification of genes by BoE/RPKM. Ovals near axes X denote approximate positions of nucleosomes upstream (Nuc−2, Nuc−1) and downstream (Nuc+1) TSS.

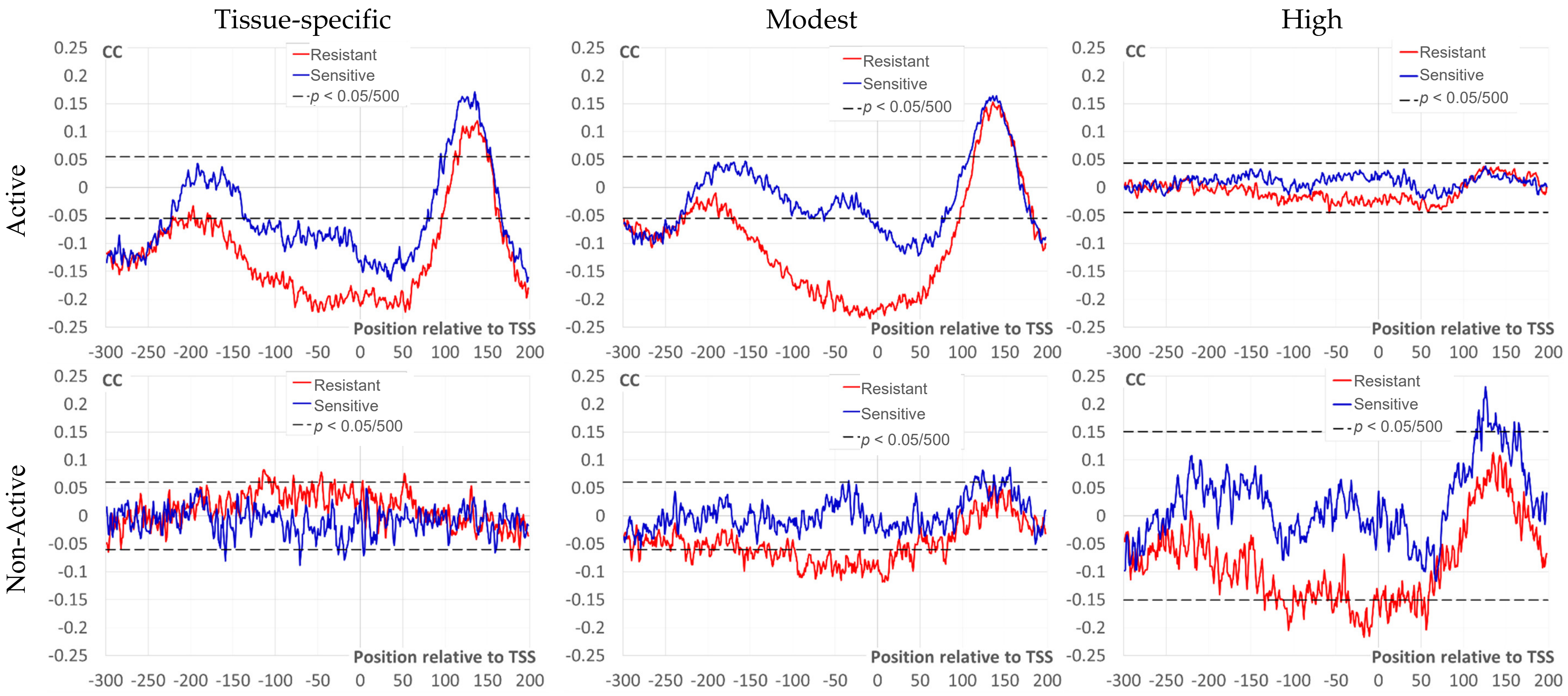

Figure S5. Kendall's rank correlation coefficients between the transcription level (RPKM) and the number of nucleosome sites. The top/bottom rows denote datasets compiles from genes referred to domains of Active (Yellow or Red) / not-Active (Green, Blue or Black) chromatin states [33]. The left, middle and right columns mark intervals of the expression breadth (BoE). Axes X – position relative to TSS, axes Y – the moving mean over a window of 3 nt for the correlation coefficient. Red/blue lines denote resistant/sensitive sites. Dashed black lines mark the threshold of Bonferroni corrected *p*-value for correlation coefficient ($p < 0.05/500$).

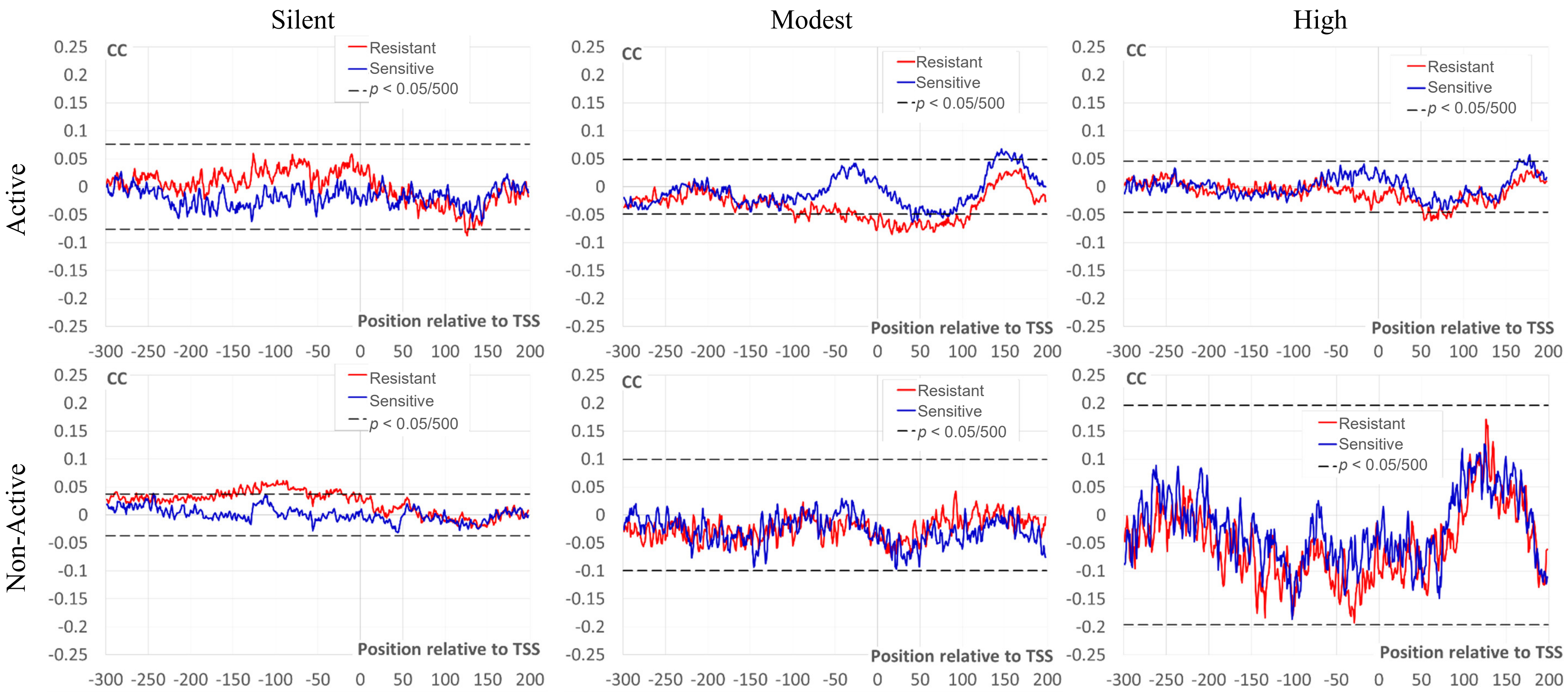

Figure S6. Kendall's rank correlation coefficients between the breadth of expression (BoE) and the number of nucleosome sites. The top/bottom rows denote datasets compiles from genes referred to domains of Active (Yellow or Red) / (Green, Blue or Black) chromatin states [33]. The left, middle and right columns mark intervals of the transcription level (RPKM). Axes X – position relative to TSS, axes Y – the moving mean over a window of 3 nt for the correlation coefficient. Red/blue lines denote resistant/sensitive sites. Dashed black lines mark the threshold of Bonferroni corrected p-value for correlation coefficient ($p < 0.05/500$).
